# Advantages of Repeated Low Dose against Single High Dose of Kainate in C57BL/6J Mouse Model of Status Epilepticus: Behavioral and Electroencephalographic Studies

**DOI:** 10.1371/journal.pone.0096622

**Published:** 2014-05-06

**Authors:** Karen Tse, Sreekanth Puttachary, Edward Beamer, Graeme J. Sills, Thimmasettappa Thippeswamy

**Affiliations:** 1 Institute of Ageing and Chronic Disease, University of Liverpool, Liverpool, United Kingdom; 2 Department of Biomedical Science, College of Veterinary Medicine, Iowa State University, Ames, Iowa, United States of America; 3 Institute of Translational Medicine, University of Liverpool, Liverpool, United Kingdom; University G. D'Annunzio, Italy

## Abstract

A refined kainate (KA) C57BL/6J mouse model of *status epilepticus* (SE) using a repeated low dose (RLD) of KA (5 mg/kg, intraperitoneal; at 30 min intervals) was compared with the established single high dose (SHD) of KA (20 mg/kg, intraperitoneal) model. In the RLD group, increased duration of convulsive motor seizures (CMS, Racine scale stage ≥3) with a significant reduction in mortality from 21% to 6% and decreased variability in seizure severity between animals/batches were observed when compared to the SHD group. There was a significant increase in the percentage of animals that reached stage-5 seizures (65% versus 96%) in the RLD group. Integrated real-time video-EEG analysis of both groups, using NeuroScore software, revealed stage-specific spikes and power spectral density characteristics. When the seizures progressed from non-convulsive seizures (NCS, stage 1–2) to CMS (stage 3–5), the delta power decreased which was followed by an increase in gamma and beta power. A transient increase in alpha and sigma power marked the transition from NCS to CMS with characteristic ‘high frequency trigger’ spikes on the EEG, which had no behavioral expression. During SE the spike rate was higher in the RLD group than in the SHD group. Overall these results confirm that RLD of KA is a more robust and consistent mouse model of SE than the SHD of KA mouse model.

## Introduction

Experimental animal models of seizures developed over many decades, have undergone numerous modifications in an effort to find the most appropriate preclinical model for screening antiepileptic drugs (AEDs), and secondarily to reduce and refine use of animals for *in vivo* experiments [1]–[8]. Despite the development of numerous new AEDs and the introduction of a variety of animal models of drug resistant epilepsy that supposedly mirror the clinical condition, the percentage of epileptic patients refractory to currently available AEDs still remains greater than 30% [9].

Rat models of seizure or epilepsy induced by chemoconvulsants such as the glutamate analogue, kainate (KA) and the cholinergic receptor agonist, pilocarpine have been commonly used for many years. Rats produce fairly consistent *status epilepticus* (SE) [6], [10], [11] with limited mortality rates (<15–20%) in most models [12]–[16]. However, over the past two decades, chemoconvulsant mouse models of seizure and acquired epilepsy have begun to emerge with the intention of utilizing transgenic mice in epilepsy research. The most commonly used background strain of mouse for this purpose is C57BL/6J [17]–[20]. C57BL/6J mice are known to exhibit low sensitivity to chemoconvulsant induced seizures [21]–[25].

Some studies have suggested that C57BL/6J mice show inconsistent seizure response to the same dose of KA, given systemically, even amongst animals derived from the same inbred source or supplier [23], [26]–[27]. Previous studies have demonstrated that C57BL/6J inbred mice are genetically resistant to KA-induced neurotoxicity and epileptogenesis [23], [24],[28]–[32]. The genetic factor and unpredictable seizure threshold for KA between batches of C57BL/6J mice might have contributed to increased mortality rate due to severity of convulsive motor seizures (CMS) during SE. From our preliminary experiments we found that in some batches of mice there was a high mortality at 20 mg/kg, while in the other batches high mortality was also observed at 10 mg/kg despite the source of KA and its formulation, and the route of administration were being the same. To overcome these disadvantages, we tested a repeated low dose (RLD) of KA in C57BL/6J mice, titrated according to the development of epileptic behavior (5 mg/kg, i.p. at 30 min intervals), and compared this with mice that received a single high dose (SHD) of KA (20 mg/kg; i.p). In the present study, we demonstrate the advantages of RLD over SHD model on mortality rate, behavioral seizures and electrographic seizure quality during the SE. Further, using integrated video-EEG NeuroScore software, we have identified stage-specific spikes and power spectrum characteristics for each stage of the behavioral seizure, and for the transition from non-convulsive seizures (NCS) to CMS in both groups.

## Materials and Methods

### Animal source and ethics statement

Experiments were performed at two sites; University of Liverpool (UoL), UK and Iowa State University (ISU), USA. C57BL/6J male mice, 10–12 weeks old, were purchased from Charles Rivers, UK or The Jackson Laboratory, ME, USA and maintained in the Biomedical Services Unit at UoL or Laboratory of Animal Resources at ISU. The mice were maintained under controlled environmental conditions (19°C–23°C, 12 h light: 12 h dark), with *ad libitum* access to food and water. All experiments were carried out in strict accordance with the Animals (Scientific Procedures) Act 1986 approved by the Secretary of State, Home Office, UK (project license no. 40/3401) and Institutional Animal Care and Use Committee, ISU, USA (protocol no. 10-12-7446-MR). All surgeries were performed under isoflurane anesthesia and all efforts were made to minimize discomfort and pain to animals.

### KA treatment and experimental groups

KA (Abcam, UK/USA) was prepared fresh in sterile distilled water at a concentration of 2 mg/ml. Based on previous studies in our laboratory [33], we considered a single dose of 20 mg/kg of KA via intraperitoneal (i.p.) route to be a suitable method for inducing CMS without very high levels of mortality. For RLD method, a low dose of 5 mg/kg of KA per injection every 30 min was given i.p. until the onset of Racine scale stage-5 seizures. The behavior of all animals were video recorded throughout the experimental period. Behavioral seizures were classified based on modified Racine scale as: Stage-1, absence-like immobility; Stage-2, hunching with facial or manual automatisms; Stage-3, rearing with facial or manual automatisms and forelimb clonus; Stage-4, repeated rearing with continuous forelimb clonus and falling; and Stage-5, generalized tonic clonic convulsions with lateral recumbence or jumping and wild running followed by generalized convulsions. Seizure stages 1 and 2 were regarded as NCS while, seizure stages 3 and higher were considered as CMS [34]. A supplementary movie clip showing CMS stage-3 to -5 can be found online (Video S1). Based on subtle behavioral and EEG characteristics, we have subdivided the stage-3 into stage-3A and -3B, which are described in the Results section.

One hundred and six mice were used for Racine scale scoring behavioral experiments after KA injections. A total of 10 mice died during SE; five out of 24 mice from the SHD group and 5 out of 82 mice from the RLD group. Two hours after the first episode of stage-5 seizure in RLD group or ≥ stage-3 seizure in SHD group, the mice were treated with diazepam (10 mg/kg, i.m). All mice were euthanized with an overdose of pentobarbital sodium (100 mg/kg, i.p.) at the end of the experiments and the brain tissues were archived.

### Video-EEG recording

Eighteen mice were subcutaneously implanted with a telemetry device (Physiotel and Multiplus ETA-F20, Data Science International, MN, USA) 8–10 days prior to KA treatment. Bilateral burr holes were made, 2.5 mm caudal to Bregma and 2 mm lateral to the midline, on each hemisphere and the electrodes were inserted into the burr holes in contact with the dura mater. The burr holes were closed by dental cement and the skin flaps were closed by skin clips. The detailed surgical procedure for implanting the electrodes and the transmitter has been described previously [33]. The RPC receiver pads, placed individually below each recording cage, transmitted the EEG, movement activity and the body temperature information via data exchange matrix to the Windows PC. The real-time video and EEG recording was captured simultaneously using DSI integrated video-EEG acquisition software (DATAquest ART, DSI, USA). The EEG recordings and the activity counts data obtained were analyzed using NeuroScore (DSI) software.

### Quantification of behavioral seizures

Video recordings from SHD and RLD groups were used to score behavioral seizures on modified Racine scale at 5 min epochs as previously described [33]. The persons scoring the episodes were unaware of the treatment groups. In each epoch, the highest seizure stage reached was considered. For example in a 5 min epoch, if stage-1 and -2 seizures occurred during the first 3 min followed by a 2 min stage-3 seizure, then the highest stage considered for behavioral scoring was CMS stage-3. This way, the seizure severity was calculated as cumulative seizure severity score (mean ± standard error) for the duration of SE based on the total amount of time spent in CMS from the time they reached first CMS to the time they received diazepam. The more severe the seizures during the SE, the higher the score. In the present study the SE refers to the 2 h duration starting from the first onset of CMS (stage≥3 for SHD group or stage-5 for RLD group) after administering KA to the time-point they received diazepam. Student's unpaired t-test was used to compare the mortality rate, duration of CMS and stage-5 seizures alone between the SHD and RLD groups using GraphPad Prism software. To compare the mortality rate between SHD and RLD groups, the mice that died were taken as “0” and survived as “1”. To compare the number of animals that reached stage-5 seizures in SHD and RLD groups, the mice that did not reach stage-5 was taken as “0” and the mice that reached were taken as “1”. The spike rate between SHD and RLD groups were compared using two-way ANOVA.

### Quantification of electrographic seizures

**Figure 1:**
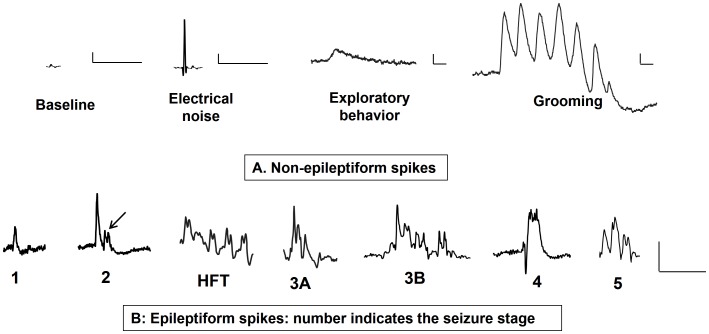
Individual spike characteristics on EEG. The baseline non-epileptiform spike and artifact spikes are shown in the top panel (A). The bottom panel (B) represents epileptiform spikes at different stages of seizures. Baseline spike, stage-1 and stage-2 epileptiform spikes are simple while, high frequency trigger (HFT), stage-3, -4 and -5 were complex. Stage-1 epileptiform spikes were similar to baseline spikes but had higher amplitude. Stage-2 epileptiform spikes had higher amplitude than stage-1. As stage-2 progressed towards CMS the spikes became complex with mini HFT-like spikes (indicated by the arrow). Several small spikes at the peak of the stage-4 spike gave a characteristic appearance of a paint brush. Stage-5 epileptiform spikes had a low amplitude when compared to the stage-4 spikes but had a combination of stage-2 to-4 spikes. Scale for all: y-axis 300 µV and x-axis 300 ms.

A minimum of 24 h baseline video-EEG was recorded for each mouse before the KA treatment. The recordings were continued to observe changes in EEG responses during KA administration. All the post-KA responses were normalized against the baseline from the same mouse. The artifacts in the raw EEG traces (electrical noise, exploratory behavior and grooming) were manually identified and excluded from the analyses. The EEG spikes that corresponded to each behavioral Racine seizure stage during a seizure, after KA administration, were regarded as epileptiform spikes (Fig. 1). In order to differentiate epileptiform spikes from the normal baseline spikes or from spikes due to electrical or mechanical artifacts, we analyzed individual spike characteristics such as amplitude, duration, frequency, inter-spike intervals (Fig. 1) and the activity counts (per min) during this period. In the EEG recordings, the epileptiform spike characteristics for each seizure stage was compared between SHD and RLD groups. The activity monitor module in the implanted telemetry device in the mouse detected the areas of high activity such as exploratory movements, grooming and seizures. Based on these, the activity threshold for CMS was set at 0.1 Hz and the activity was recorded as counts/min.

The raw EEG signal was divided into 10 s epochs, after manually excluding artifacts, and subjected to Fast Fourier Transform (FFT) to generate power bands. The signal component consisting of various frequencies were split into individual power bands corresponding to delta (δ, 0.5–4 Hz), theta (θ, 4–8 Hz), alpha (α, 8–12 Hz), sigma (Σ, 12–16 Hz), beta (β, 16–24 Hz) and gamma (γ, 24–80 Hz) [35], [36], [37]. The power in different spectra altered depending on the stage of a seizure episode and the power in some bands increased as the seizure progressed from stage-1 to -5. The mean ± standard error values pooled from 10 s epochs for parameters such as spike counts, amplitude and power band (mV^2^) were compared between SHD and RLD groups using Student's unpaired t-tests.

## Results

### RLD of KA reduces mortality rate and increases the number of animals that reach stage-5 seizures when compared with SHD

**Figure 2:**
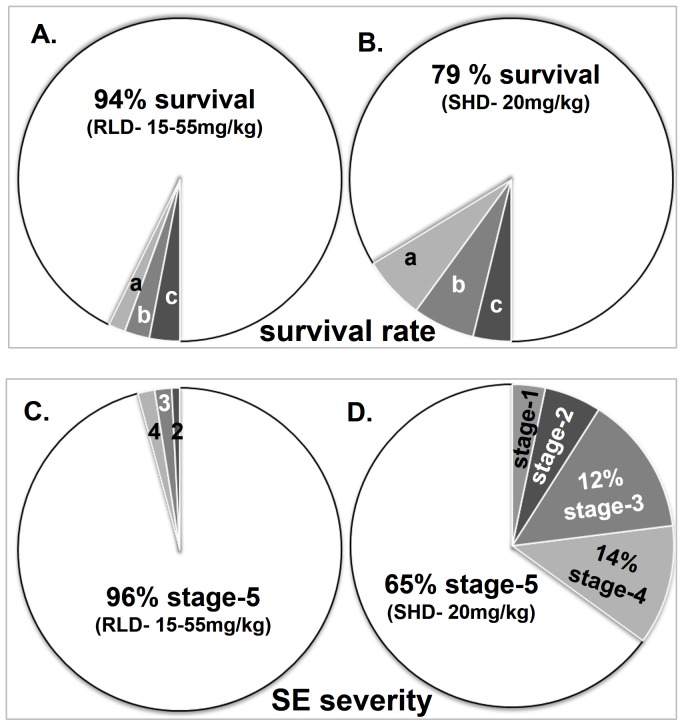
The effects of RLD and SHD of KA on mortality rate and severity of behavioral SE. RLD of KA ranging from 15–55 mg/kg (3–11 doses of 5 mg/kg at 30 min intervals, i.p.,) reduced mortality rate (A, B) and increased the percentage of animals that reached stage-5 seizures. The RLD method reduced inter-animal variability during different stages of SE (C, D). The lower case letters in pie charts A and B indicate mortality (a) during the SE, (b) soon after diazepam or (c) soon after KA injection (<10 min). The numbers in pie charts C and D indicate the stages of the seizures. N = 82 for RLD group; five mice died during SE after reaching stage-5 seizures, three mice did not reach stage-5 seizure, all mice except one reached stage-3 seizures; N = 24 for SHD group, five mice died during SE after reaching stage-5 seizures, eight mice did not reach stage-5, and 2 mice did not reach stage-3. p = 0.029 for mortality and p = 0.001 for SE.

All animals that received KA either as a SHD or as a RLD showed behavioral and electrographic seizures. The severity of seizures was classified according to the modified Racine scale [38] ranging from stage 1–5 that was previously described for C57BL/6J mouse in our publication [33]. In the present study, for RLD group, the first onset of stage-5 seizure was considered as the starting point of SE since 96% of mice reached stage-5 seizure (Fig. 2). For SHD group, the first onset of CMS-3 was considered as the starting point of SE since only 65% reached stage-5 seizure, however 91% of mice reached stage-3 (Fig. 2). In both groups, SE was terminated 2 h later which was considered as the end point of SE.

**Figure 3:**
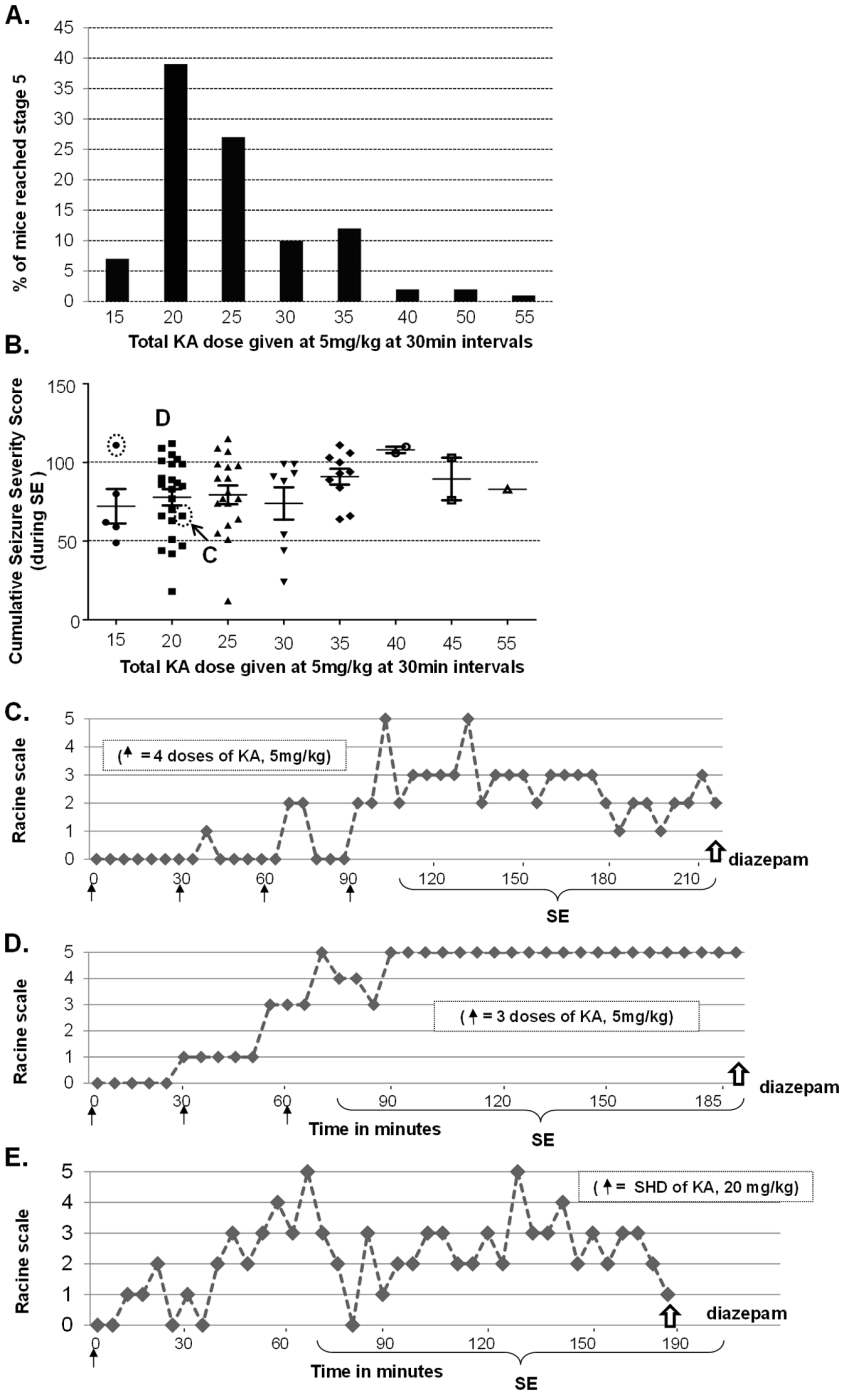
SE response over time to various doses of KA given at 5/kg at 30 min intervals and to a single dose of 20 mg/kg. A: A total of 82 mice were used for RLD of KA. About 95% of the animals reached stage-5 seizures with ≤35 mg/kg. **B**: Cumulative Seizure severity score for the 2 h duration of SE between the first stage-5 (RLD) seizure and diazepam administration. Irrespective of the total dose of KA, the vast majority of animals stayed at stage ≥3 seizures after the first stage-5 seizure. Each marker represents a mouse that had achieved SE at a given RLD of KA. C–D: Represent SE response to RLD of KA over time for an individual mouse (circled and indicated by C and D in Fig. 3B). There was no correlation between the total dose of KA received and seizure severity or the earliest time-point at which animals reached stage-5 seizures. E: SE response of mouse to a SHD of KA at 20 mg/kg over time. This mouse had a fewer CMS during the 2 h period of SE after the onset of the first stage-5 seizure.

**Figure 4:**
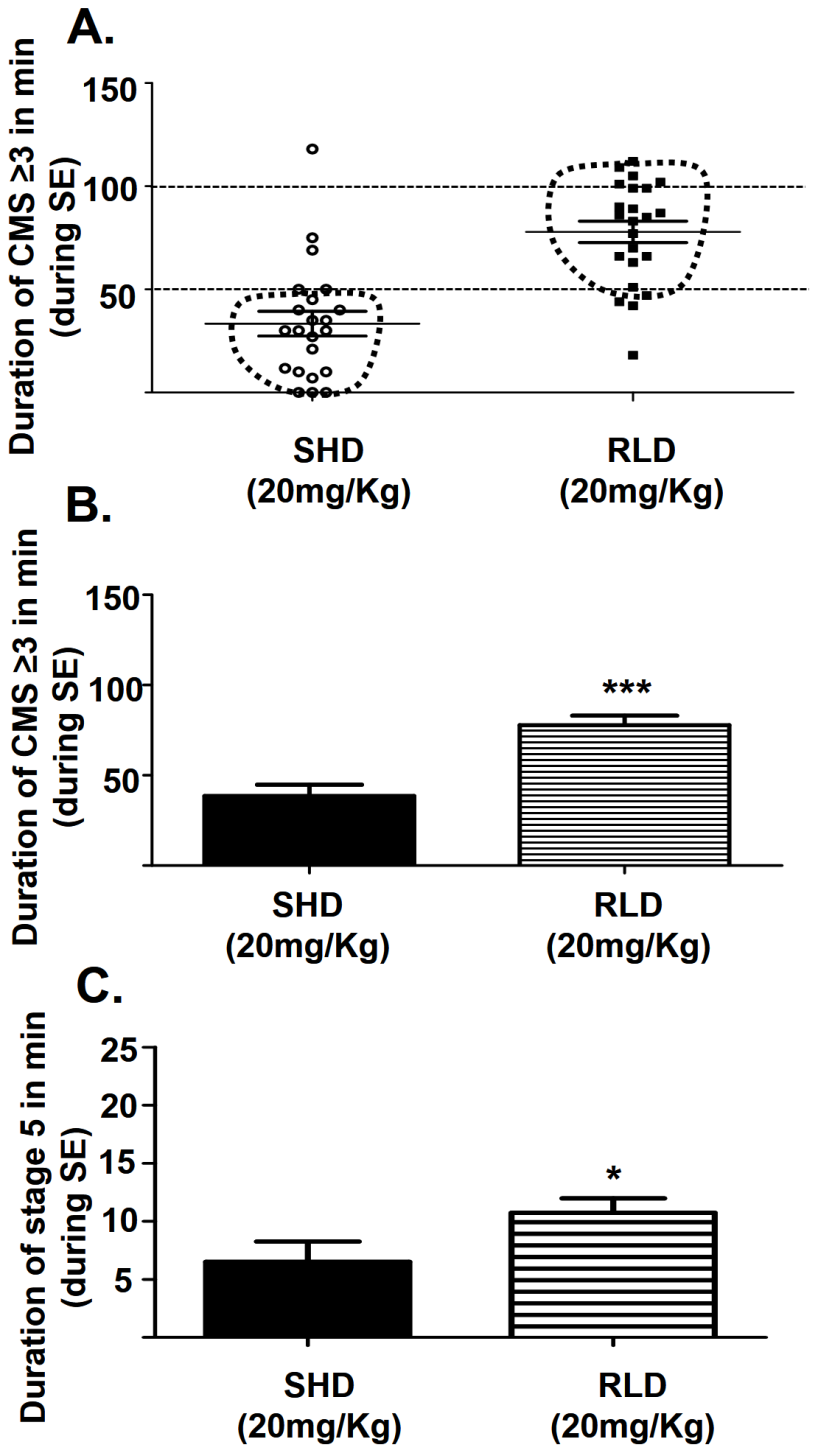
Seizure severity (total amount of time spent in CMS) comparison between SHD and RLD at 20 mg/kg (total dose in RLD or a single dose in SHD group). A–C: Suggests that the RLD method produces consistently severe seizures when compared to SHD. Each point on graph A represents an individual mouse at a specific CMS stage. B: Histogram comparing the mean ± standard error of seizure severity in SHD and RLD groups (both groups received a total of 20 mg/kg KA) based on the total amount of time spent in CMS during the 2 h SE period from the time the mice showed first CMS-3 (SHD) or CMS-5 (RLD) to the time they received diazepam C: Comparison of the amount of time spent in stage-5 alone between the two groups revealed that the RLD group had more stage-5 seizures than the SHD group (n = 19 for SHD; n = 33 for RLD; *p = 0.049, *** p<0.0001).

RLD of KA when given as 5 mg/kg at 30 min intervals resulted in a significant lower mortality rate (6% versus 21%; p = 0.029, unpaired t-test) than in a SHD of KA (20 mg/kg) (Fig 2A–B). This is despite the RLD group often receiving up to 35 mg/kg more KA than the SHD group. Importantly, there was a significant increase in the percentage of animals that reached stage-5 seizures in the RLD group when compared to the SHD group (96% versus 65%; p = 0.001, unpaired t-test; Fig 2C–D). In the RLD group, 76% of the animals reached stage-5 seizures with ≤25 mg/kg and the rest between 30 and 55 mg/kg of KA (Fig 3A). The majority of RLD group animals remained at stage ≥3 more frequently once they reached stage-5 (Fig. 3B–D) while in the SHD group it varied from stage-1 to stage-5 (Fig. 3E). A supplementary movie clip showing CMS stage-3 to -5 can be found online (Video S1). The RLD group of animals that received the same amount of KA as SHD group (20 mg/kg) spent twice the total amount of time at stage ≥3 (p<0.0001, unpaired t-test) or stage-5 alone (p = 0.049, unpaired t-test) during the SE than the SHD group (Fig. 4A–C). Interestingly, in the RLD group there was no correlation between the total dose of KA received and seizure severity or the earliest time-point at which stage-5 was reached (Fig. 3C–D). Overall, RLD of KA regimen reduced wastage of animals by 46% i.e., 15% due to difference in mortality rate and 31% due to those that did not reach stage-5 seizures in SHD group.

From quantitative perspective, in the present study, SE is quantitatively defined as the ‘total amount of time spent in CMS ≥3’ once they reach first CMS- 5 (RLD) or CMS-3 (SHD) to the time-point when diazepam was administered (which is typically calculated from 2 h duration of SE). This is represented as cumulative seizure severity score in figure 3B for individual mouse in RLD group that received various amounts of KA to achieve SE. We have also compared the quantitative SE i.e., the total amount of time spent in CMS ≥3 during the 2 h SE, between the SHD and RLD groups that received the same amount of KA (20 mg/kg). This is represented for individual mouse in figure 4A and for the groups in figure 4B.

### Individual spike characteristics, spiking rate and EEG signature for different stages of behavioral seizures in RLD and SHD group

**Figure 5:**
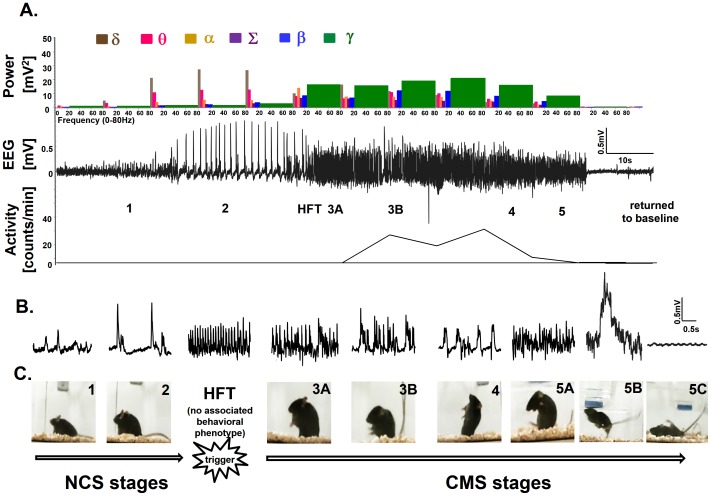
An example of an EEG trace (for 2 min) showing different stages of seizure episodes, induced by KA in C57BL/6J mouse, correlating with power spectrum, activity level and real-time behavioral seizures captured from video-EEG recording. A: EEG trace in the middle shows changes in the electrical activity as the seizure severity progressed from NCS to CMS over time. A brief HFT on the EEG, which had no behavioral counterpart, preceded the CMS. Different stages of behavioral seizures are shown by photographs (C). Magnified 2 second traces (B) representing EEG signatures for each stage of behavioral seizure. The histograms at the top panel in ‘A’ represent power bands. The baseline power of all bands was less than 5 mV^2^ and the amplitude of the baseline was <200 µV. As the seizure progressed from NCS to CMS, the power bands, and especially gamma power, increased (shown in green). Gamma power band increased after HFT, peaked at stage-3B, and started declining in stage-4 and -5 before returning to the baseline. Activity counts (per min), shown below the EEG trace, increased from stage-3A onwards and peaked in stage-3B and -4. Activity counts reduced in stage-5 when the mice were recumbent or showed generalized rigidity but, increased when the mice displayed jumping behavior. A higher delta band was found to be the hallmark of stage-2 seizures. Reduction in the peaked delta band was characteristic when NCS progressed to CMS. HFT was characterized by a peak of alpha and sigma bands. A transient increase in theta band marked the transition from stage-3A to -3B. Gamma and beta bands peaked in stage-3B and declined slowly in stage-4 and -5 before reaching the baseline.

**Figure 6:**
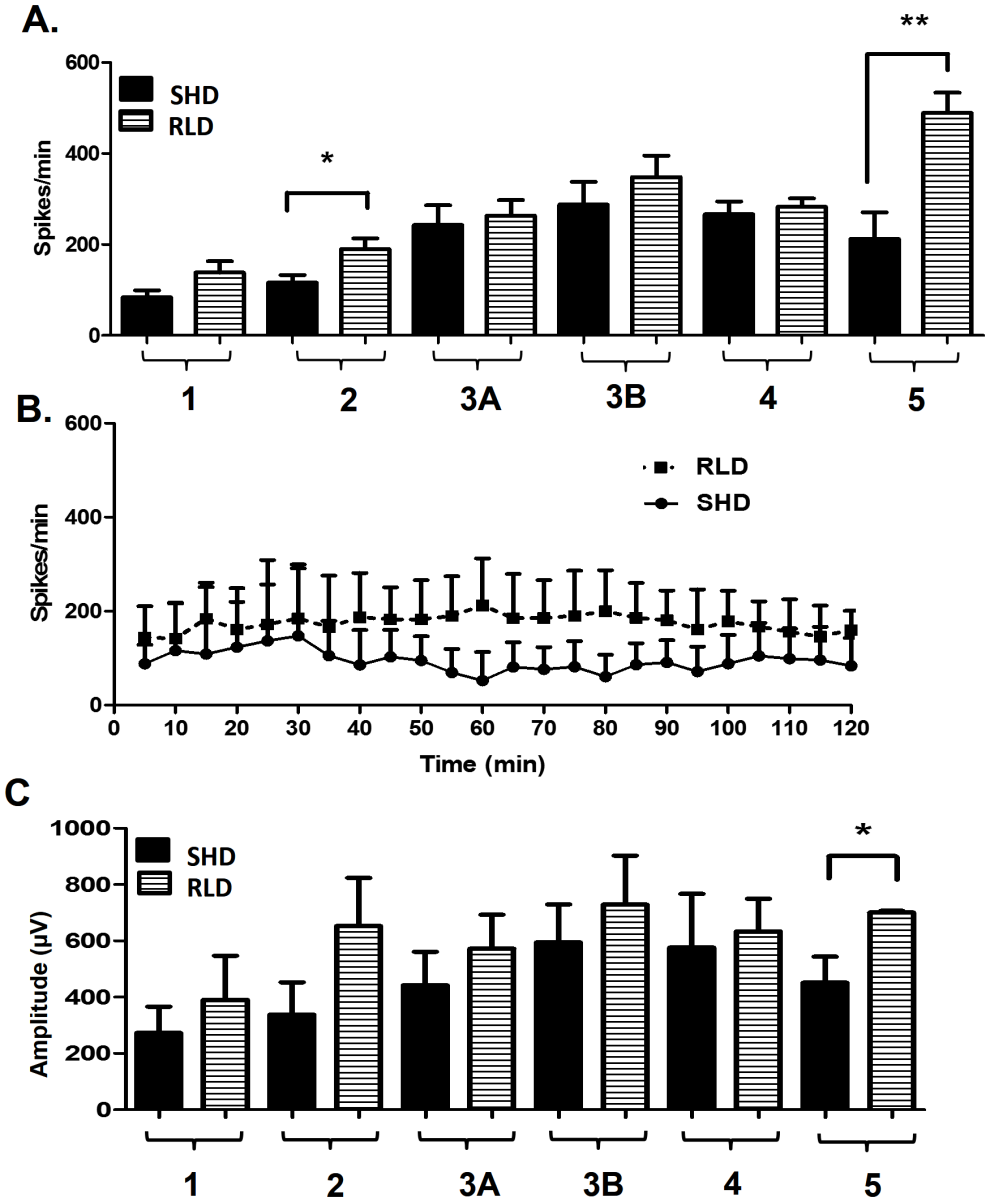
The RLD group had a higher spike count (per min) than the SHD group. Spike counts were higher across all stages of seizures (A) or in the total spike count during the entire 2 h duration of SE following RLD (B). The increase in spike counts was significantly greater during stage-2 (p = 0.021) and stage-5 (p = 0.0012) seizures in the RLD group than in the SHD group (A). The mean values of spike rate shown at 5 min epochs revealed a significant increase in the RLD group than in the SHD group (two way ANOVA, p = 0.0001, n = 9 each). **C**: The spike amplitude during the 2 h SE was greater in stage-3B ≥ stage-2 > stage-3A ≥ stage-4 ≥ stage-5 > stage-1. There was a significant increase in spike amplitude for stage-5 seizures in the RLD group (p = 0.024, n = 9 each, unpaired t-test).

We detected stage-specific epileptiform spiking characteristics from the EEG traces for different stages of behavioral seizures during the SE, which were confirmed by real-time video recordings using the NeuroScore software (Fig. 5). The epileptiform spikes were differentiated from artifact spikes based on amplitude, duration, interspike interval and spike rate (spike frequency per minute) (Fig. 1, Fig. 6; Table 1). Power spectrum characteristics and activity counts for each seizure stage were also identified (Fig. 5; Table 1).

**Table 1:** Stage-specific spike, power band characteristics and phenotypic correlates in C57BL/6J mouse treated with KA.

	EEG Spike Characteristics				Power Band Characteristics (power, mV^2^ in 10 s epochs)						Phenotypic correlates	
Stages	Amplitude (µV)	Duration of spike (ms)	Inter-spike interval (ms)	Frequency (/min)	Delta	Theta	Alpha	Sigma	Beta	Gamma	Activity (counts/min)	Behavior
**1**	200–600	100–200	400–1400	20–35	8–12	4–7	0.7–1.5	0.3–0.9	0.2–0.5	0.4–0.8	0	Freezing, Staring
**2**	200–1500	100–400	450–1500	40–60	25–50	5–15	3–5	1–3	1–3	1–2	0	Hunched rocking with stiff tail
**H F T**	300–1000	20–40	25–60	120–200	5–10	4–8	10–20	8–16	8–15	15–90	0	No specific phenotypic correlate
**3a**	300–700	50–150	100–600	40–60	10–15	6–12	8–15	8–13	6–12	15–70	0–8	Rearing, forelimbs lifted
**3b**	400–900	200–500	180–320	30–60	10–20	15–30	8–14	5–9	10–20	18–90	20–120	Forelimbs clonus, raised tail
**4**	400–700	200–400	200–800	30–70	5–20	15–20	5–10	4–8	8–15	15–50	20–80	Rearing and falling
**5**	300–700	50–200	50–200	80–140	8–15	5–15	4–8	3–7	5–12	8–30	30–60	Generalized with forelimb and hind limb clonus

There were no differences between SHD and RLD groups in stage-specific spikes and the corresponding power band characteristics. The data presented here is from an RLD group.

**Foot Note:** The lower end numbers in amplitude, duration and spike rate, and the upper end numbers in inter-spike intervals represent the period from KA injection/s to the first stage-5 seizure.

The artifact spike due to electrical interference had an amplitude of more than 2 mV with the duration of <20 ms for each spike. These spikes were observed on EEG as occasional spikes or in clusters, and produced no activity counts. However, when these spikes occurred as a cluster, it increased all power bands within the 10 s epoch, with the power ranging from 200 to 2000 mV^2^. Such artifacts were identified and excluded from the analysis. Spikes due to mechanical movement such as exploratory behavior showed a slow spike as shown in Figure 1 with activity counts up to 80/min with no change in the power spectrum. EEG for grooming was characterized by a slow amplitude spike containing series of oscillating spikes with no change in activity counts, however, increased delta (up to 700 mV^2^), theta (up to 100 mV^2^) and alpha (up to 10 mV^2^) power bands in 10 s epochs. The other power bands were unaffected by grooming behavior.

A baseline spike is a non-epileptiform spike with a low amplitude <200 µV and with no change in the power spectrum. Baseline spikes and, stage-1 and stage-2 epileptiform spikes were simple spikes while, high frequency trigger (HFT) and, stage-3, -4 and -5 were complex spikes (Fig.1, Fig. 5). Stage-2 epileptiform spikes had higher amplitude than stage-1. As stage-2 progressed towards CMS, the spikes became complex with mini HFT-like spikes (indicated by an arrow in Fig. 1). These mini-HFT spikes later became isolated and represented as high frequency clusters on EEG which marked the transition from stage-2 to -3A (Fig.5). Several small spikes at the peak of the stage-4 epileptiform spike gave a characteristic appearance of a ‘paint brush’ (Fig.1). Stage-5 epileptiform spikes had a low amplitude when compared to the stage-2 to -4 spikes (Fig.1). The details of stage-1 to -5 epileptiform spikes characteristics are given in Table 1. There were no differences with respect to stage-specific spike characteristics between SHD and RLD groups.

As previously described, NCS included stage-1 and -2 seizures. Stage-1 behavioral seizure was represented on EEG as stage-1 epileptiform spikes. The stage-2 epileptiform spikes resembled the periodic epileptiform discharges (PEDS) in both SHD and RLD groups that were similar to the pattern number 2 that we had described previously [33]. We have identified a third pattern of epileptiform spikes, HFT, on the EEG trace which preceded the CMS (Fig. 5) in both RLD and SHD groups. Interestingly, this short HFT period had no behavioral seizure characteristics. The HFT spikes had an amplitude of 300–1000 µV with a short duration of 20–40 ms. They had an inter-spike interval of 25–60 ms with a frequency of 120–200spikes/min (Fig. 5; Table 1).

We introduce a subclass under CMS stage-3 based on subtle behavioral changes, which were correlated with EEG characteristics during the SE. This is a further modification to our previously described modified Racine scale for behavioral seizures [33]. Stage-3A was characterized by rearing with facial/manual automatism but no forelimb clonus. Stage-3B represented rearing continuous with facial/manual automatism and continuous forelimb clonus (piano playing-like posture) with Straub tail. The epileptiform spikes characteristics for stage-3A and -3B are given in Table 1 and Figure1 and 5. The stage-4 behavioral seizure showed repeated rearing with continuous forelimb clonus and frequent falling which corresponded to the spikes on the EEG that had an amplitude of 400 to 700 µV with a spike duration of 200–300 ms. There were no obvious differences in stage-3A, -3B and -4 spiking rate and amplitude between the SHD and RLD groups (Fig. 6A, 6C). Stage-5 seizures were manifested by generalized tonic clonic convulsions with lateral recumbence or jumping and wild running followed by generalized convulsions. The stage-5 spike clusters contained a combination of stage-2 to -4 spikes (Fig. 1B). When the mice showed jumping behavior during stage-5 seizure, there was a corresponding “baseline shoot” for >1.5 s on EEG (Fig. 5C). As the stage-5 seizure progressed, the rigidity corresponded to a low amplitude spikes that were smaller than the baseline on the EEG (Fig.5C). The mortality during SE in the SHD group was more frequently preceded by recumbent and generalized rigidity during stage-5 seizures. However, in the RLD group the vast majority of the mice recovered from this behavior.

### RLD group showed higher spike rate than the SHD group during the SE

Overall, the spike rate (spikes/min) was greater in the RLD group than in the SHD group in all stages of seizures. The stage-2 and stage-5 epileptiform spike rates were significantly higher in the RLD group than in the SHD group (Fig. 6A; p = 0.021 for stage-2, p = 0.0012 for stage-5). Total spike counts in 5 min epochs (as spike/min) throughout the SE were also significantly increased in the RLD group (Fig. 6B, n = 9 each group, p<0.0001, F = 107.6, two way ANOVA with 1 and 384 degrees of freedom). In stage-5, there was also a significant increase in amplitude (p = 0.024) in the RLD group when compared with the SHD group (Fig. 6C).

In the present study, the quantitative SE for SHD mice had an average duration of behavioral CMS ≥3 for ≥25 min (Fig. 4B) with an average spike rate of ≥252/min, while RLD group had an average duration of behavioral CMS ≥3 for ≥60 min (Fig. 4B) with an average spike rate of ≥346/min. The average spike rates for SHD and RLD groups were calculated by pooling the number of stage-specific spikes/min during stage-3A to -5 as shown in figure 5 and 6A.

### The power bands characteristics on EEG for different stages of behavioral seizures

We determined power band spectrum for frequencies ranging from 0.5 to 80 Hz based on stage-specific EEG characteristics and the corresponding increased activity counts in 10 s epochs. The power band spectrum consisted of δ, θ, α, Σ, β and γ frequencies across different stages of seizures (Fig. 5, Table 1). As the seizure stages progressed from stage-1 to -5, the EEG patterns with increase in power bands began to emerge (Fig. 5). A magnified 2 min trace of a seizure episode shows stage-specific spike characteristics (amplitude, duration and inter-spike intervals). A corresponding power bands at 10 s epochs above the EEG trace and activity counts below the EEG trace across different stages of seizures are also shown (Fig. 5).

**Figure 7:**
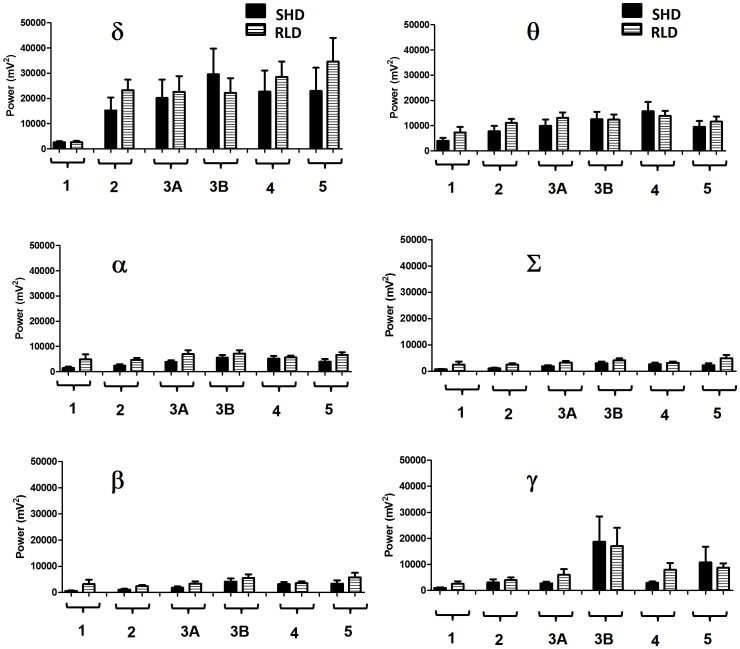
Comparison of cumulative power (mV^2^ in 10 s epochs) in the EEG during 2 h SE at different stages of seizures between SHD and RLD. The baseline power of these bands during the entire 2^2^ (data not shown). The cumulative power bands of δ, θ, α, Σ, β, and γ, frequencies increased progressively from NCS to CMS in both SHD and RLD groups. The overall power of delta, and to some extent theta except in stage-5, was higher in both SHD and RLD groups when compared to all other bands as the seizures progressed from stage-1. There were no significant changes in alpha, beta and sigma power bands at different stages of seizures, between SHD and RLD groups, although the trend was that stage-3A,-3B and stage-5 showed a marginal increase in power in the RLD group.

It is important to note that when seizures progressed from NCS to CMS, there was a visible increase in power in different bands in the EEG trace, especially power in the gamma band (Fig. 5). The gamma power increased after HFT, reached its peak during stage-3B and declined in stage-4 and -5 before reaching the baseline (Fig. 5). Higher delta and theta power was the hallmark of stage-2 spikes (PEDS). A reduction of peaked delta power and an increase in beta and gamma power marked the progression from NCS to CMS. The HFT pattern marked this transition which was characterized by a brief peak in alpha and sigma power. A transient increase in theta power marked the transition between stage-3A and -3B. Both beta and gamma power peaked in stage-3B and declined marginally in stage-4 and -5 before reaching the baseline (Fig. 5). There were no significant differences between the SHD and RLD groups in terms of power bands at different stages of seizures during SE (Fig. 7).

Activity counts (per min) increased from stage-3A and peaked in stage-3B and stage-4 (Fig. 5). Activity counts reduced in stage-5 when the mice were recumbent or during generalized rigidity, but increased when the mice showed jumping behavior during stage-5 seizures.

## Discussion

We have demonstrated the advantages of a RLD of KA to induce SE in C57B/6J mice, titrated according to the development of epileptic behavior and terminating SE with diazepam 2 h after the first expression of stage-5 seizures. We assessed this method, and compared it with conventional SHD method, by quantifying behavioral and electrographic indicators of SE severity as described previously [33]. RLD method could reliably induce SE with a prolonged period of repeated stage ≥3 seizures which was confirmed by an increased frequency of epileptiform spiking during the SE when compared to the SHD group. We have also identified stage-specific epileptiform spikes and power spectrum characteristics in EEG which were validated by integrated real-time video-EEG recordings and activity counts. A RLD of KA reduced the mortality rate from 21% to 6% when compared with the SHD and the percentage of animals that reached stage-5 seizures was increased from 65% in the SHD group to 96% in the RLD group. C57BL/6J mice have previously been reported as being less sensitive to chemoconvulsants [21]–[25] and vary in mortality rate between mice obtained from different sources [39]. Our results from RLD of KA, when compared to the SHD method, in C57BL/6J mouse model reduces variability between batches/sources and produces a robust and consistent mouse model of SE.

C57BL/6J mice are more resistant to the acute effects of KA-induced toxicity, given systemically, when compared to other inbred strains and show varied seizure responses to the same dose of KA [2], [23], [27], [28], [40], [41]. This variability might contribute to an unpredicted mortality rate. This is one of the major concerns for researchers who use C57BL/6J mouse as an acute seizure model in epilepsy research. Moreover, the majority of transgenic mice are developed on this genetic background [18], [42] and therefore it is becoming an increasingly important area of research to develop an appropriate C57BL/6J mouse model [2], [3], [43], [44] or to refine the existing models, which has been done in rats [10], [16], [45], [46]. Very recently, Loscher group in Germany have reported a C57BL/6 substrain of mouse which is significantly more susceptible to SE induction than any other C57BL/6 substrain [3] including the strain that we have been using for some time, C57BL/6J [33]. When compared to conventional SHD of KA, RLD method afforded the ability to maximize the severity and duration of SE, whilst simultaneously limiting variability and associated mortality as seen in other models [46], [47]. This is particularly important in highly invasive procedures that are expensive in cost and time, such as implanting of electrodes for recording EEG. Similar approaches have previously been used in rats to induce SE [10], [46], [47]. By administering KA by RLD method, titrated according to behavior, we demonstrate that a highly consistent period of SE with a prolonged period of repeated stage ≥3 seizures can be achieved in C57BL/6J mice. These findings, with respect to severity and duration of SE, are similar to those reported using a RLD of KA in rats by other groups [10], [34], [46]. These research groups administered KA systemically at a dose of 5 mg/kg per hour until animals showed class IV-V (equivalent to stage-4/5 in the present study). Glien et al [47] also found similar results with a RLD induction method using pilocarpine. Repeated KA dosing protocols have also been reported in C57BL/6J mice by other groups. McKhann et al [28] gave subcutaneous injections of 6–12 mg/kg of KA for every 60–90 min until CMS were observed. In another study, Yang et al [27] used 5 mg/kg KA in C57BL/6J mice, also subcutaneous, but at 1 h intervals. In these studies, an increase in severity and inter-individual uniformity was also reported, however, they have not been directly compared with animals receiving a single dose, and the characterization of the development of electrographic SE and power spectrum analyses was not done. Moreover, the first dose of KA was typically high in McKhann et al study, the interval between injections was longer due to subcutaneous route, and the mortality rate was 57% (C57BL/6J mice) in those studies [28]. This high mortality rate could be due to prolonged SE or due to the fact that SE was not terminated pharmacologically. With respect to the effect of the prolonged duration of SE, our behavioral findings are in close agreement with Yang et al. [27] whose treatment regimen was similar to the present study including SE termination (albeit using pentobarbitone rather than diazepam and a different route of administration of KA). However by comparison, our method yielded much lower mortality rate i.e., 6% as opposed to 27% and 57% in other studies [27], [28]. In our study, in addition to recording behavioral SE, we have also quantified electrographic seizures, validated them in relation to real-time video recordings of behavioral SE and made direct comparisons between those induced by RLD and SHD of KA. It is interesting to note that in RLD group, the stage-2 spikes (PEDS-like spikes) and stage-5 spike frequency was higher than in the SHD group. These findings also validate RLD as the most robust method of SE. PEDS pattern is emerging as a candidate for predictor of EEG seizures [53]. Staley et al [54] proposed that the correlation of spikes and epilepsy has long been recognized and forms the basis for the diagnostic use of the EEG. The spike characteristics identified in the present study may support the prognostic significance of spikes and their frequency in experimental models of epilepsy and this approach has been utilized in human temporal lobe epilepsy and epilepsy-related surgeries [55]–[58]. In the present study, in addition to PEDS-like spikes we have also identified “HFT” spike pattern with no behavioral correlates, which marked the transition between NCS to CMS. Although at present there is no evidence from the literature to support this finding, like PEDS, HFT pattern may be useful as a predictor of CMS.

It has been reported that C57BL/6J mice are more resistant to KA-induced excitotoxic cell death when KA was administered intraperitoneally at a dose <30 mg/kg [23], [27], [28]. High doses of KA (>30 mg/kg) required to induce CMS caused death of CA1 pyramidal cells in C57BL/6J mice [23]. Benkovic et al [30] have also demonstrated widespread neurotoxicity following KA treatment (35 mg/kg) in C57BL/6J adult mice. KA administration disrupts blood-brain-barrier irrespective of the dose of KA used [27], [30], [48]. Recent evidence suggests that intra-hippocampal [59]–[61] or intranasal administration [62]–[63] of KA causes localized neuron death in the hippocampus. Interestingly, there are no strain differences (KA-susceptible DBA and KA-resistant C57BL/6J) in the basal level of expression of KA or AMPA receptors in hippocampus and other brain regions [26], [49]. This means that irrespective of the amount of KA administered in RLD group, the receptor density is unchanged and the overall effects of KA are due to summation of all receptor activities. It is speculative at this stage that, as the brain concentration of KA increases, following either SHD or RLD, it could activate NMDARs in addition to KA and AMPA receptors in the hippocampus [50], [54]. Experimental evidence suggests that the activation of these receptors in the hippocampal network and the entorhinal cortex generate post-synaptic potentials [51], [52]. According to Lehmkuhle et al [35], the power in the gamma band reflects the frequency range of excitatory postsynaptic potentials that would be expected to occur during seizures. In the present study, the dynamic gamma and beta power were observed in the EEG during CMS in both SHD and RLD groups. There were no statistically significant differences in any of the power spectral bands at any stages between SHD and RLD. One plausible reason is that in RLD group there were more number of stage-5 seizures which were not always associated with increased beta and gamma power in 10 s epochs and the maximum power was evident only during the stage-3B (Fig. 5), which was similar in both SHD and RLD groups. We observed higher delta and theta frequencies in stage-2 (NCS) spiking which were similar to ‘absence seizure’ rat model (WAG/RiJ) [64]–[65]. During the transition from NCS to CMS, an increase in alpha and sigma bands were observed in HFT spiking pattern in the present study, however the significance of this finding is yet to be determined in predicting spontaneous recurrent seizures in chronic epilepsy model.

In summary, RLD of KA in C57BL/6J mice decreases SE variability, reduces mortality rate and produces consistent prolonged CMS and electrographic SE when compared to the SHD of KA. Integrated video-EEG data analysis provides stage-specific spike and power spectrum characteristics that occur during SE and has great potential for facilitating screening of pharmacological interventions targeting NCS and CMS. Application of these parameters in our ongoing post-SE studies in both mouse and rat models of epilepsy provide a valuable means of identifying genuine spontaneous recurrent seizures and inter-ictal spikes. Because the C57BL/6J is the most popular background strain for genetic manipulations, the development of a more useful model of chronic epilepsy, beyond SE, would be a major step forward.

## Supporting Information

Video S1 **A movie demonstrating convulsive motor seizures stage-3 to -5 (Racine 3–5) in C57BL/6J mouse.**
(MPG)
